# Prostatic Cryoablation to Treat Lower Urinary Tract Symptoms for Benign Prostatic Hyperplasia Patients Not-Responding or Not-Amenable to Prostatic Artery Embolization

**DOI:** 10.1007/s00270-026-04485-5

**Published:** 2026-05-19

**Authors:** Leonard Paul Hortling, Peter Bischoff, Andreas Schäfer, Michael Pinkawa, Attila Kovács

**Affiliations:** 1 WEGE Klinik, Villenstraße 8, 53129 Bonn, Germany; 2 University Hospital Bonn, Venusberg Campus 1, 53127 Bonn, Germany

Up to 20% of patients with lower urinary tract symptoms (LUTS) and benign prostatic hyperplasia (BPH) do not respond to prostatic artery embolization (PAE) [1]. A minority of patients being considered for PAE are also not treated due to vascular, renal, or other reasons. Prostatic cryoablation (PCryo) is an established treatment option for the focal treatment of prostate cancer [2]. Driven by patients’ desire to receive a minimally invasive and function-preserving alternative, we investigated the feasibility and safety of PCryo for treating LUTS in patients with BPH.

We performed a single-center retrospective cohort study between October 2020 and April 2024, including 26 patients with BPH and severe LUTS (*n* = 17 non-responders to PAE; *n* = 7 extensive vascular atherosclerosis, *n* = 1 renal insufficiency, and *n* = 1 back pain unable to lie flat precluding PAE). Institutional review board approval was obtained for the retrospective data analysis, and informed consent was obtained from all patients.

**Fig. 1:**
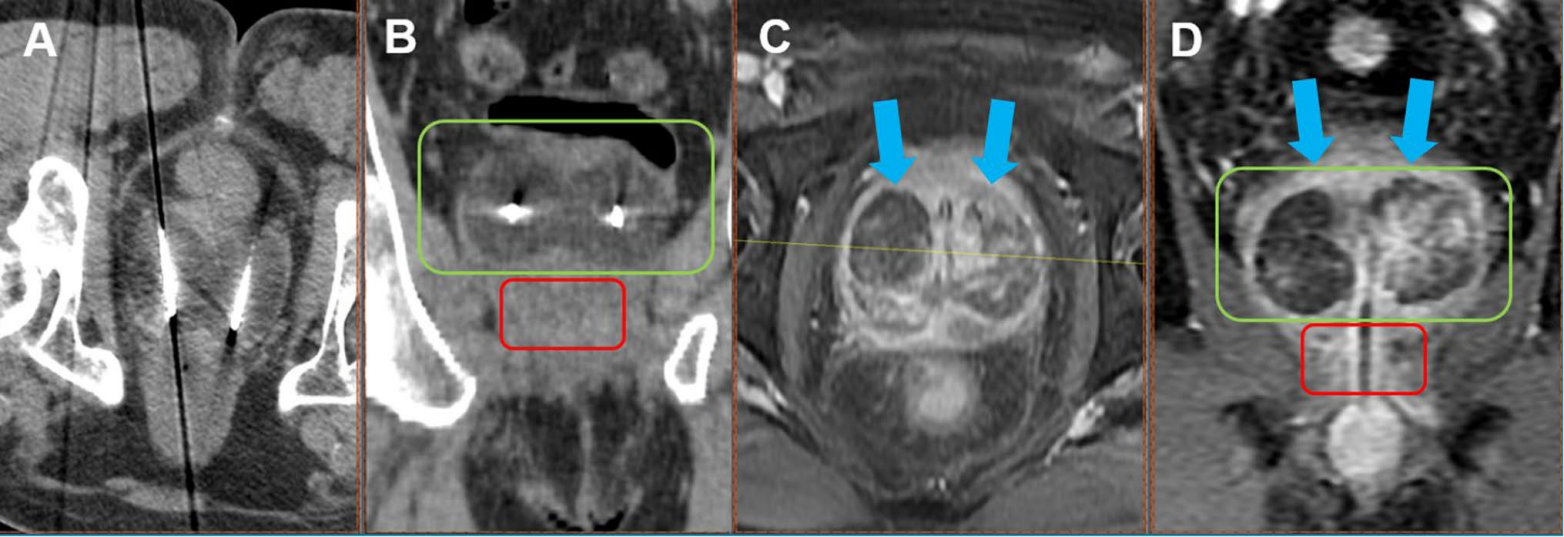
Intra- and post-procedural imaging of prostatic cryoablation. **A** Axial CT imaging demonstrates the converging course of the cryoablation probes in prone position in the right and left prostatic transition zone base. The image shows the positioning of the tip of the cryoprobes up to the ventral border of the prostate. **B** The coronal CT reformations confirmed the correct positioning of both probes at the same desired height over the base and middle transitional zone, avoiding the apex (to preserve the functionality of the external urethral sphincter). **C** Axial, T1-weighted, fat-suppressed contrast-enhanced MRI of the prostate 48 h after cryoablation. The converging ablation zones are well documented on both sides (arrows). Cryoablation remains limited to the prostate, with no extension outside the prostate capsule. **D** Coronal, T1-weighted, fat-suppressed contrast-enhanced MRI depicting converging cryoablation of the prostatic transitional zone base and middle third (arrows), with the deliberate omission of the prostate apex

A CT-guided transgluteal approach, in prone position, under low-dose midazolam sedoanalgesia with anesthetic support was used in all patients (Fig. 1A). Based on CT imaging, the puncture trajectories were defined to target the base and middle third of the transition zone of the prostate on either side (Fig. 1B green square). The course of the probes converged three-dimensionally from the outside-caudal to the inside-cranial. The puncture channel runs through the pararectal space on both sides, a fat-filled corridor bounded by the small gluteal, internal obturator, and levator ani muscles. A special feature of the pararectal corridor is that it is largely free of vascular and nerve structures; thus, from the perspective of interventionists, it represents an ideal pathway to approach the prostate [3]. Cryoprobes were inserted under CT fluoroscopic guidance. After evaluating the correct position of the active tips of the cryoprobes, the base and middle third of the prostatic transition zone were frozen under CT fluoroscopic guidance. The prostate apex was deliberately left unfrozen to avoid damage to the external urethral sphincter and associated with incontinence (Fig. 1B red square). Depending on the volume to be treated, IceForce or IcePearl probes (CX Cryoablation needles, Boston Scientific®) were used, with either an elliptical or spherical ablation volume. The median ablation volumes were: IceForce 40.4 ± 17.8 ml; IcePearl 21.6 ± 9.8 ml.

**Fig. 2:**
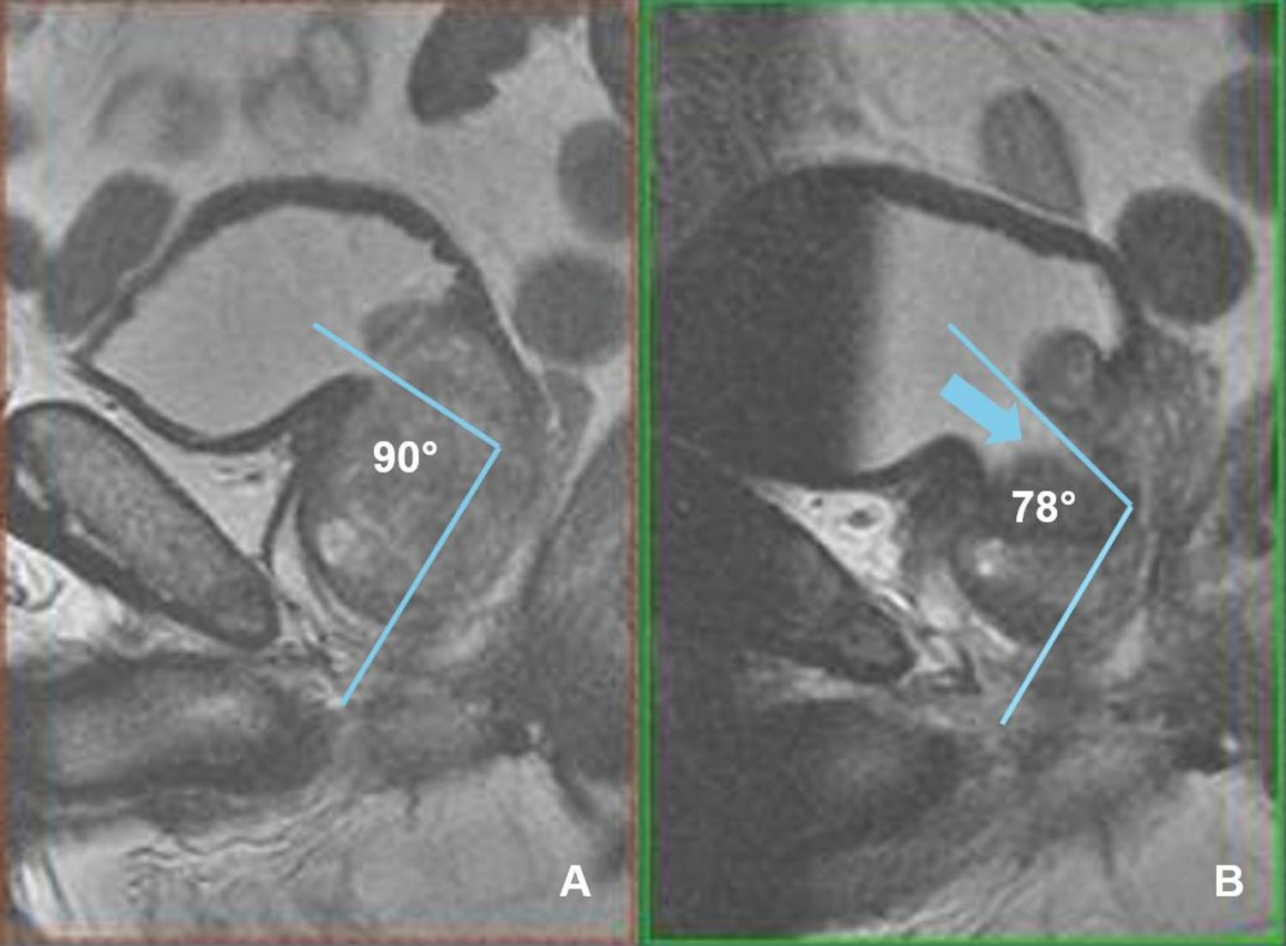
Sagittal T2-weighted image of the pelvis before (**A**) and 6 months after (**B**) prostatic cryoablation. In addition to the general reduction in prostate volume, successful opening and relief of the urinary bladder outlet can also be seen (arrow) with an improvement in the prostatic urethral angle

The freezing and thawing cycles were as follows: 5 min of freezing—5 min of active thawing—5 min of freezing—5 min of active thawing. The cryoprobes were then removed. Adverse events were recorded using the CIRSE classification of complications [4]. Technical success was verified using contrast-enhanced MRI 48 h after the procedure. The International Prostate Symptom Score/Quality of Life Score (IPSS/QoL), prostate volume (PV), intravesical prostatic protrusion (IPP), and prostatic urethral angle (PUA) were measured using MRI prior to, 2, and 6 months after PCryo. The IPP was measured in the midsagittal plane on T2-weighted MR images of the prostate as the shortest perpendicular distance between the protruded end of the prostate and the bladder base on the bladder neck in the sagittal plane. The prostatic urethral angle (PUA) was measured as the angle between the prostatic and membranous urethra in the midsagittal plane using T2-weighted MR images (Fig. 2) [1]. Statistically significant differences were set for *p* values of < 0.05.

**Fig. 3:**
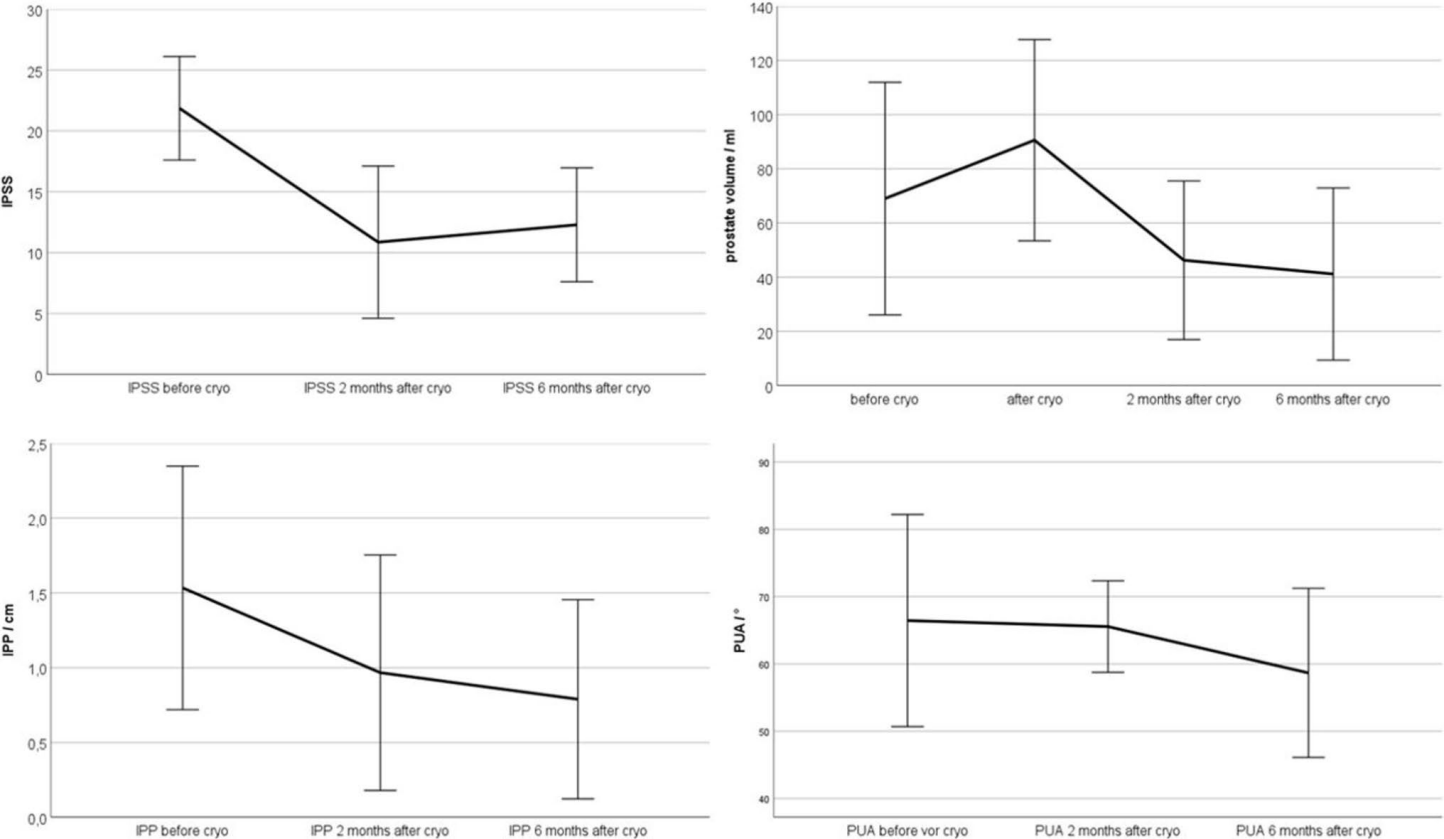
The average IPSS decreased from 21.39 ± 3.9 to 11.7 ± 5.3 (*p* = 0.011) six months after cryotherapy. The PV showed a temporary increase during the initial post-intervention phase by 72% at 48 h after ablation, which is to be interpreted as therapy-associated edematous swelling. PV subsequently decreased significantly from 63.6 ± 34.5 ml pre-cryo to 33 ± 31.7 6 months post-cryo (*p* < 0.001). Positive, though not statistically significant, changes were observed in the IPP and PUA. Both decreased in the course of 6 months from 1.4 ± 0.6 to 0.5 ± 0.7 cm (p < 0.102), and from 68.5 ± 10.4° to 58.2 ± 11.4° (*p* < 0.138), respectively

The mean patient age was 72.5 ± 9.3 years. Cryoablation was technically feasible in 100% of cases. The average frozen volume was 29.1 ± 16.2 ml. Minor complications included only mild and temporary hematuria (*n* = 18). None of the patients experienced incontinence, erectile dysfunction, or ejaculatory disorders. No major complications were observed. Six months after PCryo, the IPSS decreased from 21.39 ± 3.9 to 11.7 ± 5.3 (*p* = 0.01); the QoL improved from 5.5 ± 1 to 3 ± 1.6 (*p* = 0.17); PV decreased from 63.6 ± 34.5 to 33 ± 31.7 mL (*p* < 0.001), the IPP decreased from 1.4 ± 0.6 to 0.5 ± 0.7 cm (*p* = 0.102), and the PUA reduced from 68.5 ± 10.4° to 58.2 ± 11.4° (*p* = 0.138) (Fig. 3).

Prostatic ablation to treat LUTS/BPH has been previously performed via transperineal access (e.g., TPLA, transperineal laser ablation). Here, we provide preliminary results showing the feasibility and safety of transgluteal PCryo as an alternative option or complement to PAE. We regard the significant improvement in the IPSS—considered the gold standard for monitoring LUTS caused by BPH—as another important finding of this study. The present study has several limitations, including its single-center, single-operator, retrospective design, which limits generalizability. Uroflowmetry was not evaluated. The small sample size, lack of a control group, relatively short follow-up period (6 months), and non-significant findings for IPP and PUA are also acknowledged. Despite the results, future comparative trials with transperineal (e.g., TPLA), transurethral (e.g., TURP, HoLEP, Rezum, AquaBeam) ablation therapies of the prostate, as well as PAE are required to confirm the evidence and assess the best minimally invasive therapies for treating patients with BPH with LUTS [5].
